# p62/sequestosome 1 as a regulator of proteasome inhibitor-induced autophagy in human retinal pigment epithelial cells

**Published:** 2010-07-27

**Authors:** Johanna Viiri, Juha M. T. Hyttinen, Tuomas Ryhänen, Kirsi Rilla, Tuomas Paimela, Erkki Kuusisto, Ari Siitonen, Arto Urtti, Antero Salminen, Kai Kaarniranta

**Affiliations:** 1 Department of Ophthalmology, Institute of Clinical Medicine, University of Eastern Finland, Kuopio, Finland; 2 Department of Anatomy, Institute of Biomedicine, University of Eastern Finland, Kuopio, Finland; 3 Department of Neurology, Institute of Clinical Medicine, University of Eastern Finland, Kuopio, Finland; 4 Centre for Drug Research, Faculty of Pharmacy, University of Helsinki, Helsinki, Finland; 5 Department of Ophthalmology, Kuopio University Hospital, Kuopio, Finland

## Abstract

**Purpose:**

The pathogenesis of age-related macular degeneration involves impaired protein degradation in retinal pigment epithelial (RPE) cells. The ubiquitin-proteasome pathway and the lysosomal pathway including autophagy are the major proteolytic systems in eukaryotic cells. Prior to proteolysis, heat shock proteins (HSPs) attempt to refold stress-induced misfolded proteins and thus prevent the accumulation of cytoplasmic protein aggregates. Recently, p62/sequestosome 1 (p62) has been shown to be a key player linking the proteasomal and lysosomal clearance systems. In the present study, the functional roles of p62 and HSP70 were evaluated in conjunction with proteasome inhibitor–induced autophagy in human RPE cells (ARPE-19).

**Methods:**

The p62, HSP70, and ubiquitin protein levels and localization were analyzed by western blotting and immunofluorescense. Confocal and transmission electron microscopy were used to detect cellular organelles and to evaluate the morphological changes. The p62 and HSP70 levels were modulated using RNA interference and overexpression techniques. Cell viability was measured by colorimetric assay.

**Results:**

Proteasome inhibition evoked the accumulation of perinuclear aggregates that strongly colocalized with p62 and HSP70. The p62 perinuclear accumulation was time- and concentration-dependent after MG-132 proteasome inhibitor loading. The silencing of p62, rather than Hsp70, evoked suppression of autophagy, when related to decreased LC3-II levels after bafilomycin treatment. In addition, the p62 silencing decreased the ubiquitination level of the perinuclear aggregates. Recently, we showed that *hsp70* mRNA depletion increased cell death in ARPE-19 cells. Here, we demonstrate that *p62* mRNA silencing has similar effects on cellular viability.

**Conclusions:**

Our findings open new avenues for understanding the mechanisms of proteolytic processes in retinal cells, and could be useful in the development of novel therapies targeting p62 and HSP70.

## Introduction

Age-related macular degeneration (AMD) is the leading cause of blindness of elderly people in the developed countries. The disease affects the macula, which is located in the central area of the retina. Primarily, AMD is characterized by degeneration of the macular retinal pigment epithelial (RPE) cells [1]. The RPE cells take care of the health of rods and cones. Therefore, the degeneration and cell death of RPE cells cause secondary adverse effects on the neural cells, ultimately leading to visual loss. Chronic oxidative stress and inflammation are key factors evoking RPE degeneration and promoting the AMD process [2,3]. One hallmark of AMD is the accumulation of lysosomal lipofuscins, and extracellular drusens between RPE cells and Bruch’s membrane. This cargo is a clear evidence of a disturbance in the cellular protein clearance system in aged RPE cells [1]. Recently, it has been documented that in human AMD donor samples and in RPE cell cultures, there are increased levels of autophagic markers and decreased lysosomal activity [4-7].

Eukaryotic cells have two major proteolytic systems for the clearance of proteins: the first is the ubiquitin-proteasome pathway and the second is the vesicle-dependent lysosomal pathway [8]. The ubiquitin-proteasome system recognizes and selectively degrades oxidatively damaged soluble proteins that have not been successfully repaired by molecular chaperones such as heat shock proteins (HSPs) [1]. Prior to proteolysis, these proteins are tagged with a small polypeptide called ubiquitin [9,10]. It has been demonstrated that proteasomes are suppressed in RPE cells during the aging process [11]. Autophagy, which can be subdivided into macroautophagy, microautophagy, and chaperone-mediated autophagy, is part of the lysosomal proteolytic mechanism [12]. The autophagy usually shares its proteolytic burden with proteasomes during normal cellular homeostasis and protein clearance in response to cellular stress and the aging process [1,13,14]. The lysosomes receive material for degradation from different intra- and extracellular mechanisms. Quantitatively, autophagy is the major process that delivers substrates to the lysosomal compartment for degradation [15]. Recently, there has been an increased general interest in understanding the interactions of proteasomes and autophagy in protein clearance.

p62/sequestosome 1 (SQSTM 1) has been shown to be a missing link combining the functions of the proteasomal and lysosomal clearance systems [16]. The p62 is a scaffold protein with multiple roles in cell signaling, receptor internalization, and protein turnover. It is also known as ORCA, lckBP, A170, or ZIP. The p62 was first identified as a phosphorylation-independent ligand of the lck Src-like tyrosine kinase (lckBP), and independently as an oxidative-stress upregulated protein (A170) and as a ligand of atypical PKC (ZIP) or RIP kinase [17]. It has been reported to be regulator of inflammation, neurogenesis, osteoclastogenesis, adipogenesis, and T-cell differentiation. One of the most interesting functions of p62 is the regulation of transcription factor NF-kappa-B, which is the master regulator of innate immunity and aging [14,17-21]. The p62 protein is commonly found in inclusion bodies containing polyubiquitinated protein aggregates [22]. Ubiquitinated protein aggregates are p62 positive in several neurodegenerative diseases such as in Parkinson, Alzheimer, and Huntington’s diseases [23-26].

Furthermore, p62 serves as a shuttling factor for the delivery of ubiquitinated substrates to the proteasome [20,27]. It has a ubiquitin-associated domain at its C-terminus, enabling noncovalent binding to ubiquitin or ubiquitinated substrate proteins [20]. At first, p62 proteins are polymerized with each other via the Phox and Bem1p (PB1) domain in the N-terminus. Subsequently, TNF receptor associated factor 6 (TRAF6) is attached to the TRAF6 binding site of p62 with its ubiquitin chains in restricted situations. These branched chains (K48, K63, employing lysine, K) are then transferred from TRAF6 to substrate proteins, which finally interact with ubiquitin-associated domain of p62. These complexes are then transported to proteasomal degradation, where the N-terminal PB1 domain of p62 binds to the proteasome. Alternatively, ubiquitinated complexes are shuttled to lysosomes for autophagocytic degradation [17,20,21,28]. The proposed mechanism for the sorting of p62-ubiquitin-substrate protein complexes to autophagic degradation is the interaction with microtubule-associated protein 1 light chain 3 (LC3). The specific autophagy effector LC3 is one of the several autophagy-related genes (Atgs). Subsequently, p62 interacts directly with LC3 in the LC3 recognition sequence domain. If there are mutations in p62 such that it lacks the LC3 binding site, this causes the formation of inclusion bodies, similarly as during autophagy-deficiency [21,29-31].

In this study, the regulatory role of p62 ubiquitin adaptor/scaffold protein and HSP70 molecular chaperone were evaluated in proteasome inhibitor-induced macroautophagy in human RPE cells (ARPE-19).

## Methods

### Cell culture and treatments

ARPE-19 cells were obtained from American Type Culture Collection. The cells were grown to confluency in a humidified 10% CO_2_ atmosphere at 37 °C in Dulbecco’s Modified Eagle Medium:F12 (1:1; Gibco, Invitrogen, Carlsbad, CA), including 10% inactivated fetal bovine serum (Hyclone, Logan, UT), 100 units/ml penicillin, 100 µg/ml streptomycin and 2 mM L-glutamine (all three from Lonza, Verviers, Belgium). For proteasome inhibition, the cells were exposed to 250 nM–5 µM MG-132 proteasome inhibitor (Calbiochem, San Diego, CA) for 24 h. The lysosomes were made alkaline by the addition of 50 nM bafilomycin A1 (Sigma-Aldrich, Steinheim, Germany).

### Western blotting

For western blotting, whole cell extracts (15–20 µg of protein) were run in 10% or 15% sodium dodecyl sulfate PAGE (PAGE; SDS–PAGE) gels and then wet-blotted to nitrocellulose membranes (Amersham, Pittsburgh, PA). The membranes were blocked for 1 h in 3% fat-free dry milk in 0.3% Tween-20/phosphate buffered saline (0.9% PBS; 2 mM NaH_2_PO_4_xH_2_O, 0.15 M NaCl, 10 mM NaH_2_PO_4_xH_2_O) at room temperature (RT). Thereafter, the membranes were incubated for 1 h at RT with rabbit polyclonal ubiquitin antibody (cat. no. Z0458; DakoCytomation, Glostrup, Denmark), mouse monoclonal SQSTM1 (p62) antibody (cat. no. sc-28359; Santa Cruz Biotechnology, Santa Cruz, CA), or rabbit polyclonal LC3 antibody (cat. no. AP1802a; Abgent, San Diego, CA). Primary antibodies were diluted (1:500, 1:2,000, or 1:250, respectively) in 0.5% BSA (BSA) in 0.3% Tween-20/PBS. After washing 3X for 10 min with 0.3% Tween-20/PBS, the membranes were incubated for 1 h at RT with horseradish peroxidase-conjugated antirabbit IgG or antimouse IgG antibodies (GE Healthcare, Little Chalfont, Buckinghamshire, UK). The secondary antibodies were diluted (for ubiquitin 1:15,000, for p62 1:25,000, and for LC3 1:5,000) in 3% fat-free dry milk in 0.3% Tween-20/PBS. Before detection, all of the membranes were washed as before. Protein-antibody-complexes were detected with an enhanced chemiluminescent assays for horseradish peroxidase (Millipore, Billerica, MA).

### Immunofluorescence

The cells were fixed with 4% paraformaldehyde in PBS for 10 min at RT. To determine the localization of p62 and ubiquitin, the permeabilization was achieved with 0.2% Triton X-100 in PBS for 15 min at 4 °C. The samples were blocked with 3% fat-free dry milk in 0.05% Tween/TBS for 30 min at RT. The antibodies (rabbit polyclonal ubiquitin 1:80 and mouse monoclonal SQSTM1 1:500, see previous chapter) were diluted in 0.05% Tween/TBS and incubated for one hour with samples at RT. After washing 3× for 5 min with PBS, the samples were incubated with secondary antirabbit or antimouse (Alexa Fluor 488/Alexa Fluor 568; Invitrogen, Carlsbad, CA) antibody diluted to 1:1,000 in 2% BSA/PBS for 1 h at RT and washed as before. Nuclei were stained in all samples by incubation with Hoechst 33258 dye (0.5 µM/ml) for 5 min at RT. Images were captured using a Nikon Eclipse TE300 microscope equipped with a Nikon E995 digital camera (Nikon, Tokyo, Japan).

### pDsRed2-hp62 and pEGFP-hHSP70 fusion plasmid construction

Human *p62* (h*p62*, *SQSTM 1*; NCBI nucleotide accession number NM_003900) cDNA (cloned in pOTB7 vector) was purchased from RZPD (product no. IRAUp969A0355D6; Deutsches Ressourcenzentrum für Genomforschung GmbH, Berlin, Germany). The open reading frame (ORF) of h*p62* was further amplified with primers 5′-ATA *CTC GAG* at**A TG**G CGT CGC TCA CC3′ and 5′-TAT *AAG CTT* a**TC A**CA ACG GCG GGG GAT G-3′ containing restriction sites for XhoI and HindIII, respectively. The translation initiation and termination sites are shown in boldface. The additional bases enabling in-frame cloning are in lower case letters. The sticky ends for the amplified *p62* ORFs, as well as for the multiple cloning site of the vector pDsRed2-C1 (Clontech, Mountain View, CA), were produced with the above-mentioned restriction endonucleases (MBI Fermentas, Vilnius, Lithuania). Ligated (T4 DNA Ligase, Roche, Basel, Switzerland) DNA forming a fusion gene of DsRed2 and *p62* was transfected into competent DH5α *Escherichia coli* cells, which were prepared using the high efficiency transformation protocol of Inoue et al. [32], cultured, and purified [33]. The integrity of the construct, denominated hereafter as pDsRed2-hp62 (h for human) was determined initially by restriction endonuclease digestion analysis and finally sequencing of the junction sites and the entire inserted *p62* ORF. Details of the GFP-HSP70 construct (pEGFP-hHsp70) have been recently documented [13].

### Studying of the p62 and HSP70 colocalization in ARPE-19 cells

For colocalization studies in living cells, ARPE-19 cultures were grown on 8-well glass slides (Nunc Lab-Tek® Chambered Borosilicate Coverglasses, cat. no. 155411; Nalge Nunc, Rochester, NY). Before plating the cells, the glasses were treated with collagen Type IV (50 µg/ml in 20 mM acetic acid; Becton Dickinson, Franklin Lakes, NJ) for 1 h and rinsed with phosphate buffer and culture medium. Cells were grown for 24 h before the transfection of fluorescent plasmids to achieve 50%–70% confluency. Briefly, 500 µg of plasmid, either pEGFP-hHSP70 or pDsRed2-hp62, or both simultaneously (250 ng each), was added to diluted ExGen 500 transfection reagent (MBI Fermentas) following the manufacturer’s instructions. Briefly, 500 µg of plasmid, either pEGFP-hHSP70 or pDsRed2-hp62 or both simultaneously (250 ng+250 ng), was added to diluted ExGen 500 non-toxic cationic polymer-based transfection reagent (MBI Fermentas) following the manufacturer's instructions. Transfection was continued for 24 h in a total volume of 200 µl on each well, and subsequently proteasome inhibitor MG-132 (Calbiochem) was added to the cultures at a 5 µM concentration for 24 h.

The fluorescent fusion proteins were localized with confocal microscopy (Nikon Eclipse TE 300 microscope with a 100× oil immersion objective; Nikon). The EGFP stain was visualized at an excitation wavelength of 488 nm and emission at 525 nm. The wavelengths for monitoring DsRed2 fluorescence were 558 nm for excitation and 583 nm for emission, and for EGFP excitation and emission wavelengths were 488 nm and 525 nm, respectively.

### Attenuation of human *p62* and *hsp70* gene expression by RNA interference

**Figure 1:**
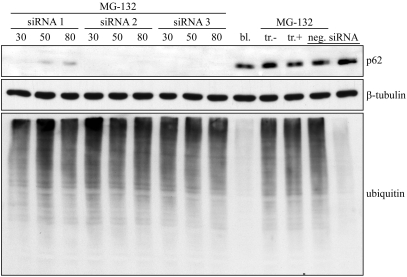
Western blotting analysis for evaluating *p62* RNA interference efficacy and measuring its effects on ubiquitin levels in ARPE-19 cells. Three different *p62* siRNAs were used with three different concentrations (30, 50, and 80 nM). The samples of *p62* siRNA were also treated with 5 µM MG-132. Bl. stands for untreated control, tr. for transfection reagent (with or without 5 µM MG-132), and negative siRNA for nonsilencing siRNA (with or without 5 µM MG-132). All exposures lasted 24 h. α-Tubulin staining was used to ensure the equal loading of proteins.

To find the effective *p62* siRNA, three different siRNAs designed for human *p62* gene (chosen siRNA for further experiments was number 3 in Figure 1, cat no. s16962) and a nonsilencing siRNA (cat. no. AM 4611) were used (Ambion, Austin, TX). These were transfected into ARPE-19 cells using siPORT™ amine transfection reagent (Ambion) following the manufacturer’s instructions. Reagent includes blend of polyamines formulated for transfection of small RNAs. The tested concentrations of all *p62* siRNAs in cell cultures were 30, 50, and 80 nM (30 nM for the negative control) and the duration of siRNA treatment was 24 h. The decrease in *p62* expression was monitored using western hybridization with mouse monoclonal p62 antibody (Santa Cruz), as described above. The silencing of the *hsp70* gene has been recently documented [13].

### Transmission electron microscopy

Cell cultures treated for 24 h with the negative control, *p62* (number 3 in Figure 1), and *hsp70* (cat. no. 16708; Ambion), siRNAs (concentration of all three 30 nM), and MG-132 (5 µM; Calbiochem), following a recovery period of 0, 24, or 48 h were prefixed with 2.5% glutaraldehyde and sodium phosphate buffer (0.1 M, pH 7.4) for 2 h at room temperature. After washing (15 min in phosphate buffer), samples were postfixed with 1% osmium tetraoxide in phosphate buffer for 1 h and then washed with phosphate buffer before normal ethanol dehydration. The samples were infiltrated and embedded in LX-112 resin (Ladd Research Industries, Burlington, VT). Polymerization was performed at 37 °C for 24 h and at 60 °C for 48 h. The sections were finally examined with a Jeol JEM-1200EX transmission electron microscope (Jeol, Tokyo, Japan; 80 kV).

### Viability staining of cells

Cellular viability was assessed with MTT assay. In the MTT assay, the ability of cells to metabolize the yellow MTT tetrazole salt (3-(4,5-dimethylthiazol-2-yl)-2,5-diphenyltetrazolium-bromide) to purple formazan is measured spectrophotometrically at 570 nm [34].

### Statistical analysis

The statistically significant differences were identified with SPSS for Windows software (v. 14; SPSS, Chicago, IL) using Mann–Whitney U-test. Variations were represented by standard deviation (n=4). p-values below 0.05 were considered significant.

## Results

**Figure 2:**
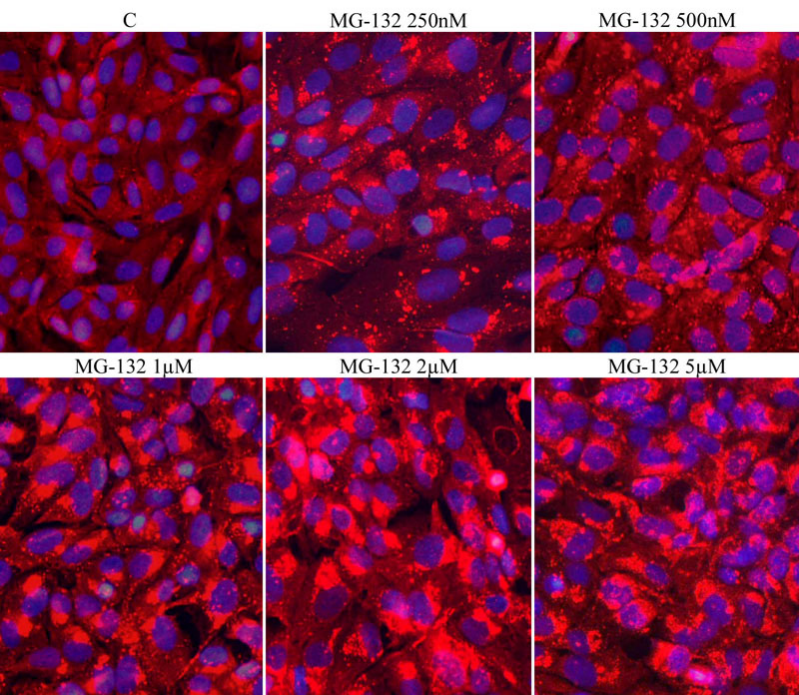
Immunofluorescence microscopy analysis of p62 (red) in ARPE-19 cells. Cells were exposed to different concentrations (250 nM–5 µM) of MG-132 for 24 h. C stands for control. Nuclei are stained with blue dye.

To study the effect of different concentrations of proteasome inhibition on p62 localization, human ARPE-19 RPE cells were either nonstressed or exposed to 250 nm – 5 µM MG-132 for 24 h. The staining of p62 was then analyzed by immunofluorescence microscopy. A clear, concentration-gradient-dependent increase in the intensity of p62 staining was seen in perinuclear space in response to the MG-132 treatments (Figure 2).

**Figure 3:**
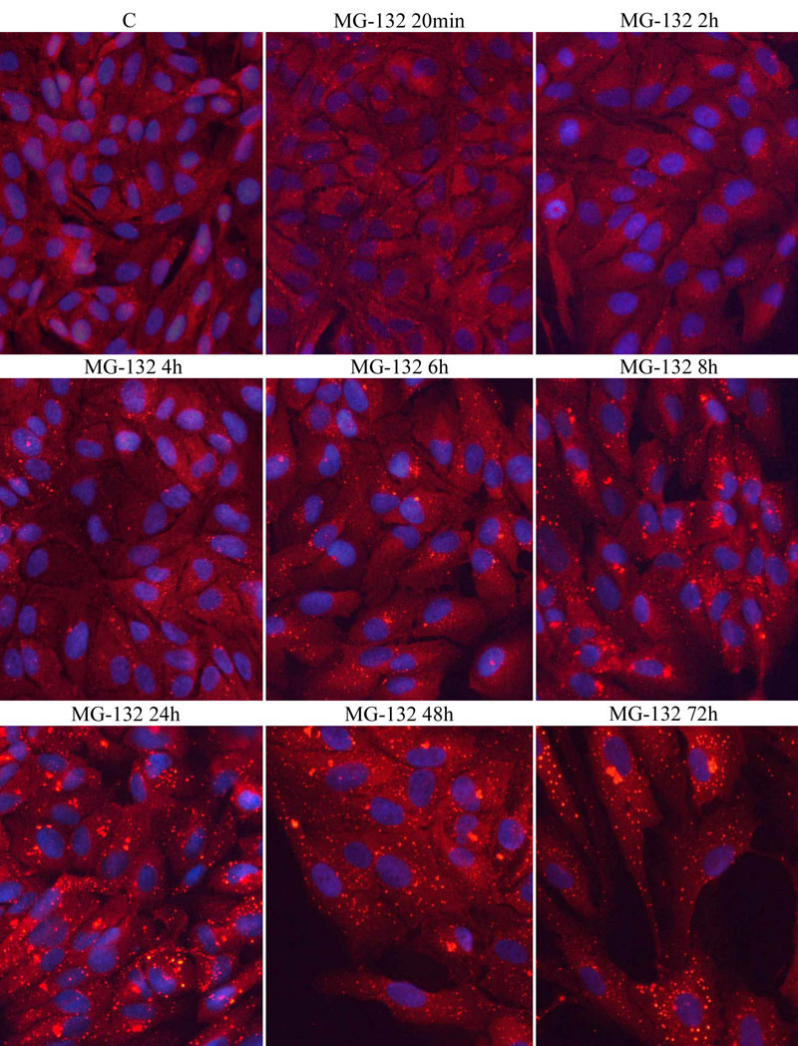
Immunofluorescence microscopy analysis of p62 (red) ARPE-19 cells. Cells were exposed to 250 nM MG-132 for different time periods (20 min–72 h). C stands for control. Nuclei are stained with blue dye.

A constant 5 µM MG-132 exposure was used to analyze p62 perinuclear accumulation during different time points from 20 min to 72 h. A clearly increased p62 staining in the perinuclear space occurred after 8 h of treatment, but an even stronger p62 response was observed at the later time points (Figure 3).

The efficacy of *p62* siRNA was analyzed with western blotting. The cells were either nonstressed or exposed to 5 µM MG-132 with or without of *p62* mRNA silencing for 24 h. Western blotting analysis revealed effective blocking of the p62 synthesis in response to the mRNA silencing when three different p62 siRNAs (siRNA 1, siRNA 2, siRNA 3) were examined with three different concentrations (30, 50, and 80 nM). For further analyses 30 nM siRNA 3 was used to suppress p62 expression (Figure 1). Total ubiquitin protein conjugate levels were accumulated to a great extent in response to MG-132 treatment. However, there was no marked difference in total ubiquitin protein conjugate levels between MG-132 treated cells or combined MG-132 treatment with *p62* mRNA silencing.

**Figure 4:**
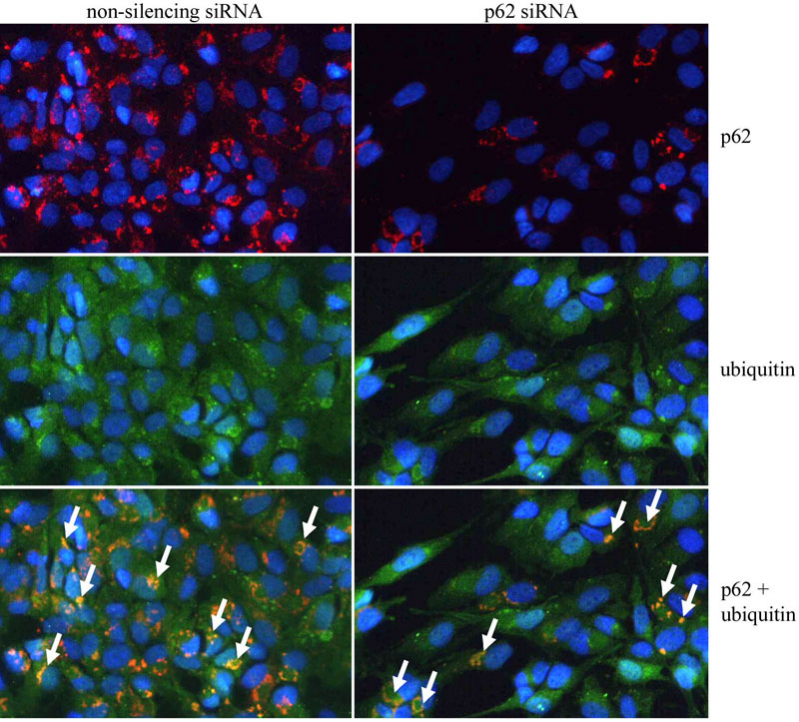
Immunofluorescence microscopy analysis of p62 (red) and ubiquitin (green) in ARPE-19 cells. Cells were exposed to 5 µM MG-132 with nonsilencing siRNA or with *p62* siRNA (both 30 nM) for 24 h. Nuclei are stained with blue dye. Arrows point to the colocalization of p62 and ubiquitin in perinuclear protein aggregates.

Since p62 is known to be a ubiquitin-binding protein, the cells were treated with nonsilencing and silencing *p62* siRNA oligonucleotides, together with 5 µM MG-132 for 24 h and then analyzed by immunofluorescence microscopy. The p62 depletion evoked a decrease in the perinuclear stainings of p62, but also in the ubiquitin protein conjugation, as found when analyzed by immunostaining (Figure 4). Note that no change in the total ubiquitin protein conjugate level was observed between MG-132 treated cells or combined MG-132 treatment with *p62* mRNA silencing (Figure 1). This reveals that p62 might regulate the localization of ubiquitin-conjugate levels rather than affecting their total amount in proteasome inhibitor–treated ARPE-19 cells.

**Figure 5:**
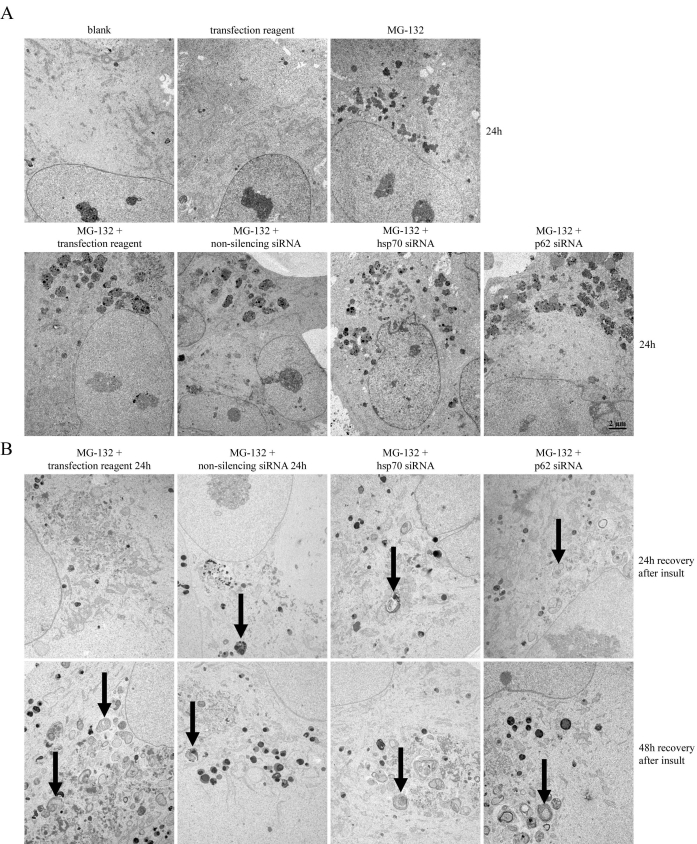
Analysis of perinuclear aggregates. **A**: Transmission electron micrographs of control ARPE-19 cells (blank), cells exposed to transfection reagent, and cells exposed to 5 µM MG-132 for 24 h with or without transfection reagent, nonsilencing siRNA, *hsp70* siRNA, or *p62* siRNA (all 30 nM). The scale bar equal to 2 μm. **B**: Transmission electron micrographs of the cells simultaneously treated with 5 µM MG-132 and the transfection reagent, nonsilencing RNA, *hsp70* siRNA, or *p62* siRNA for 24 h, and then allowed to recover in normal cell culture medium for 24 or 48 h. Arrows indicate autophagosomal structures.

The HSP70 molecular chaperone, proteasomes, and autophagy have an important regulatory role in the protein turnover of human RPE cells [13]. The role of p62 was studied in perinuclear protein aggregation and autophagy clearance by treating ARPE-19 cells with nonsilencing and silencing siRNAs for *hsp70* or *p62*. The cells were nonstressed, exposed to 5 µM MG-132 for 24 h, or exposed to 5 µM MG-132 for 24 h and allowed to recover in normal cell culture medium up to 48 h. The ultrastructure of the cells was examined using transmission electron microscopy. In control cells, no abnormalities were observed, whereas MG-132 evoked a prominent perinuclear deposit accumulation, as expected (Figure 5A). We have previously demonstrated that the accumulated material is lysosomal LAMP-1 and LAMP-2 positive [13]. Both hsp70 and p62 depletion evoked similar autophagy-like structures in perinuclear space in response to the proteasome inhibition; this was followed by a recovery in normal cell culture medium (Figure 5B).

**Figure 6:**
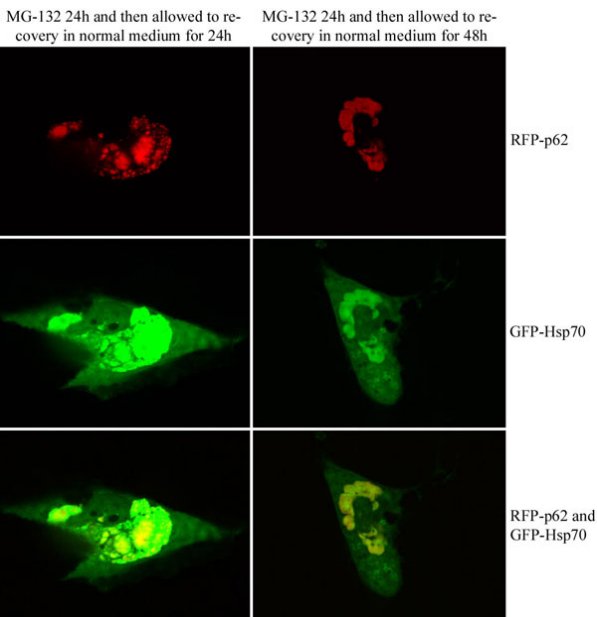
Colocalization of DsRed2-p62 and GFP-HSP70 in ARPE-19 cells, revealed by confocal microscopy analysis. Cells have been exposed to 5 µM MG-132 for 24 h.

The localization of pDsRed2-hp62 and pEGFP-HSP70 was studied by exposing ARPE-19 cultures to 5 µM MG-132 for 24 h and then allowing them to recover for either 24 or 48 h. The pDsRed2-hp62 and pEGFP-hHSP70 constructs showed that both p62 and HSP70 proteins were strongly colocalized to the same area in perinuclear deposits after proteasome inhibition (Figure 6).

**Figure 7:**
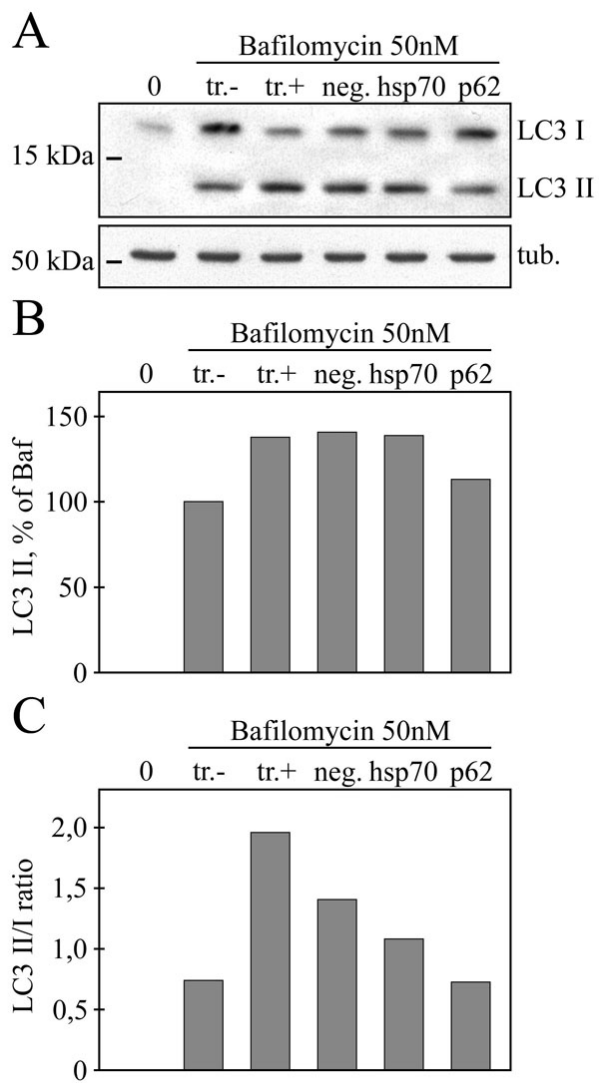
Western blotting analysis for evaluating the effects of *p62* or *hsp70* RNA interference on LC3 levels in ARPE-19 cells treated with bafilomycin. **A**: The cells were exposed simultaneously to 50 nM bafilomycin and the transfection reagent, nonsilencing RNA, *hsp70* siRNA, or *p62* siRNA for 24 h. The untreated control is represented by 0, while tr. stands for transfection reagent, negative for nonsilencing siRNA, *hsp70*, and *p62* for silencing siRNAs. α-tubulin staining was used to ensure equal loading of proteins. **B**: Normalization against α-tubulin. **C**: The ratio of a lipidated LC3-II to a nonlipidated LC3-I. Experiments were repeated two independent times.

The *p62* and *hsp70* RNA interference effects on LC3 levels, as an indicator of autophagy dynamics, were analyzed by western blotting. ARPE-19 cells were exposed to 50 nM bafilomycin simultaneously with nonsilencing siRNA, *hsp70* siRNA, or *p62* siRNA for 24 h. The silencing of *p62* mRNA decreased LC3-II accumulation and the ratio of LC3-II/I, while *hsp70* siRNA did not markedly affect the LC3-II levels (Figure 7).

**Figure 8:**
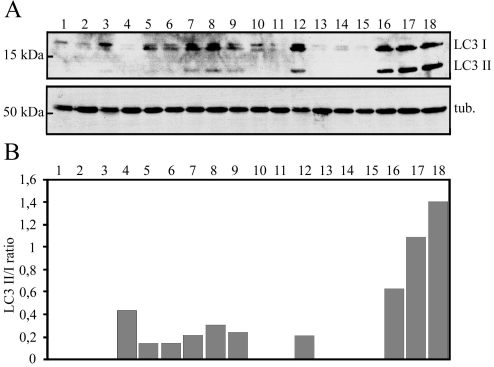
Analysis of LC3 expression levels. **A**: western blotting analysis for evaluating the effects of *p62* or *hsp70* RNA interference on LC3 levels in ARPE-19 cells treated with 5 µM MG-132 and 50 nM bafilomycin. Lane 1. control medium for 36 h; lane 2. MG-132 for 24 h + recover 6 h; lane 3. control medium for 24 h + bafilomycin for 6 h; lane 4. MG-132 + nonsilencing RNA for 24 h + recover 6 h; lane 5. MG-132 + *p62* siRNA for 24 h + recover 6 h; lane 6. MG-132 + *hsp70* siRNA for 24 h + recover 6 h; lane 7. MG-132 + nonsilencing RNA for 24 h + recover in bafilomycin for 6 h; lane 8. MG-132 + *p62* siRNA for 24 h + recover in bafilomycin for 6 h; lane 9. MG-132 + *hsp70* siRNA for 24 h + recover in bafilomycin for 6 h; lane 10. control medium for 48 h; lane 11. MG-132 for 24 h + recover 24 h; lane 12. control medium for 24 h + bafilomycin for 24 h; lane 13. MG-132 + nonsilencing RNA for 24 h + recover 24 h; lane 14. MG-132 + *p62* siRNA for 24 h + recover 24 h; lane 15. MG-132 + *hsp70* siRNA for 24 h + recover 24 h; lane 16. MG-132 + nonsilencing RNA for 24 h + recover in bafilomycin for 24 h; lane 17. MG-132 + *p62* siRNA for 24 h + recover in bafilomycin for 24 h; lane 18. MG-132 + *hsp70* siRNA for 24 h + recover in bafilomycin for 24 h. α-tubulin staining was used to ensure equal loading of proteins. **B**: The ratio of a lipidated LC3-II to a nonlipidated LC3-I. Experiments were repeated two independent times.

Our recent findings reveal that bafilomycin is a stronger autophagy inducer than MG-132 in ARPE-19 cells [13]; therefore, we also wanted to study the effects of bafilomycin in proteasome inhibitor–treated ARPE-19 cells. The cells were exposed to 5 µM MG-132 simultaneously with nonsilencing siRNA, *p62* siRNA, or *hsp70* siRNA for 24 h, and then allowing the cells to recover in normal cell culture medium or in 50 nM bafilomycin for 24 h. Bafilomycin clearly increased the LC3-II/I ratio in proteasome inhibitor–treated cells. Only a mild effect of LC3-II levels was observed in response to pure MG-132 exposure. Neither *p62* nor *hsp70* siRNA decreased the LC3-II/I ratio in cells exposed to proteasome inhibition (Figure 8). This reveals that in certain concentrations, proteasome inhibitor–induced autophagy might be independent of the p62 protein.

**Figure 9:**
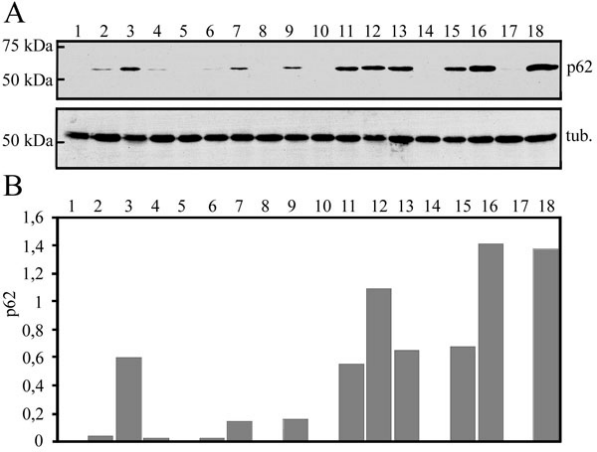
Analysis of p62 expression levels. **A**: western blotting analysis for evaluating the effects of *p62* or *hsp70* RNA interference on p62 levels in ARPE-19 cells treated with 5 µM MG-132 and 50 nM bafilomycin. Lane 1. control medium for 36 h; lane 2. MG-132 for 24 h + recover 6 h; lane 3. control medium for 24 h + bafilomycin for 6 h; lane 4. MG-132 + nonsilencing RNA for 24 h + recover 6 h; lane 5. MG-132 + *p62* siRNA for 24 h + recover 6 h; lane 6. MG-132 + *hsp70* siRNA for 24 h + recover 6 h; lane 7. MG-132 + nonsilencing RNA for 24 h + recover in bafilomycin for 6 h; lane 8. MG-132 + *p62* siRNA for 24 h + recover in bafilomycin for 6 h; lane 9. MG-132 + *hsp70* siRNA for 24 h + recover in bafilomycin for 6 h; lane 10. control medium for 48 h; lane 11. MG-132 for 24 h + recover 24 h; lane 12. control medium for 24 h + bafilomycin for 24 h; lane 13. MG-132 + nonsilencing RNA for 24 h + recover 24 h; lane 14. MG-132 + *p62* siRNA for 24 h + recover 24 h; lane 15. MG-132 + *hsp70* siRNA for 24 h + recover 24 h; lane 16. MG-132 + nonsilencing RNA for 24 h + recover in bafilomycin for 24 h; lane 17. MG-132 + *p62* siRNA for 24 h + recover in bafilomycin for 24 h; lane 18. MG-132 + *hsp70* siRNA for 24 h + recover in bafilomycin for 24 h. α-tubulin staining was used to ensure equal loading of proteins. **B**: Normalization against α-tubulin levels.

In addition to LC3, it is also possible to use p62/SQSTM1 as an autophagy marker [35]. The p62 protein serves as a link between LC3 and ubiquitinated substrates; p62 becomes incorporated into the completed autophagosome and is degraded in autolysosomes. Therefore, ARPE-19 cells were exposed to 5 µM MG-132 simultaneously with nonsilencing siRNA, *p62* siRNA, or *hsp70* siRNA for 24 h, and then the cells were allowed to recover in normal cell culture medium or 50 nM bafilomycin for either 6 or 24 h. When the cells were exposed to bafilomycin, a clear p62 accumulation was observed in western blots (Figure 9). This indicates autophagy clearance for p62 in proteasome inhibitor –treated ARPE-19 cells. Taken together, our findings reveal that there is a threshold level for p62 to regulate autophagy in ARPE-19 cells. The p62-dependent autophagy occurs in a strong bafilomycin-induced response, while a weaker autophagy induction after proteasome inhibition might be independent of p62.

**Figure 10:**
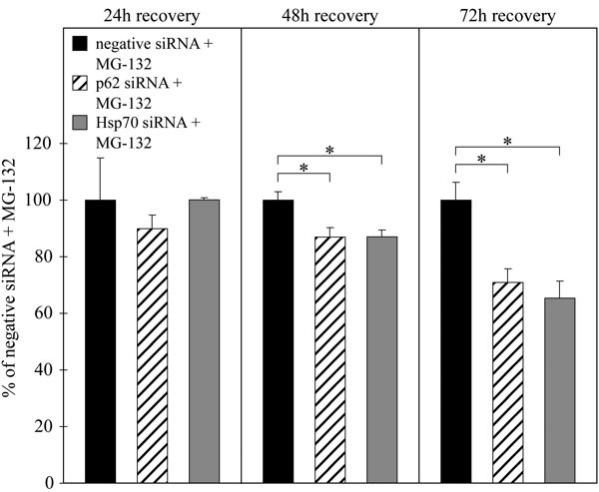
MTT assay of ARPE-19 cells. The columns represent the relative viability (negative siRNA fixed to 100%). Cells were exposed to 5 µM MG-132 with *p62* or *hsp70* siRNA for 24 h and then allowed to recover in normal cell culture medium. Variations are represented by standard deviation (n=4); p values below 0.05 (*) were considered significant in the Mann–Whitney U test.

We have recently shown [13] that HSP70 has a cytoprotective capacity in response to proteasome inhibition. In the present experiments, we wanted to compare the effect of p62 to HSP70. Thus, we treated the cells with 5 µM MG-132, subjected them to *p62* or *hsp70* mRNA silencing for 24 h, and then allowed them to recover in normal cell culture medium up to 72 h. The MTT cell viability analysis (Figure 10) showed that there is evidence of a similar cytoprotective capacity between p62 and HSP70 in proteasome inhibitor–treated ARPE-19 cells.

## Discussion

We have recently shown that HSP70, proteasomes, and autophagy work together to regulate protein turnover in human RPE cells [13]. In this study, we demonstrate that the proteasome inhibitor MG-132 evoked a strong time- and concentration-related accumulation of p62 in perinuclear lysosomal vesicles/residual bodies. Interestingly, pDsRed2-hp62 and pEGFP-hHSP70 confocal microscopy analyses revealed strong colocalization of p62 and HSP70 in the perinuclear protein aggregates after proteasome inhibition. A comparison of the *p62* mRNA and *hsp70* mRNA depletion indicates that both proteins have similar cytoprotective effects in response to proteasome inhibition in ARPE-19 cells. In contrast, the silencing of *p62* mRNA, but not *hsp70*, reduced LC3-II expression with treatment with the macroautophagy inhibitor bafilomycin. In addition, the silencing of *p62* mRNA changed ubiquitin protein conjugate localization, but did not affect total ubiquitin protein levels after proteasome inhibition. Based on our previous [13] and the present findings, we believe that both p62 and HSP70 are crucial molecules in the regulation of autophagy-linked proteolysis and cell viability in human RPE cells, but they have different functions in these cellular processes and are not regulated by each other.

The p62 was found in polyubiquitinated protein aggregates in response to proteasomal depletion [22]. Subsequently, it has been observed that p62 is expressed in many neurodegenerative diseases such as in Parkinson, Alzheimer, and Huntington’s diseases [23-26]. Interestingly, several proteins identified in the deposits occurring in Alzheimer disease have also been found in eye samples isolated from patients with AMD [36]. The p62 protein is one of the main molecules controlling the shuttling of ubiquitinated substrates to the proteasomal degradation [20,27]. At present, there is emerging evidence that the p62-ubiquitin-substrate protein complexes are being targeted to autophagic degradation [31,37]. In this paper, we demonstrate that increased accumulation of perinuclear aggregates and p62 are concentration- and time-dependent on MG-132 loading. We observed mild accumulation of LC3-II, which reveals autophagosomal activity, in response to proteasome inhibitor treatment in ARPE-19 cells. However, there was no difference in LC3-II levels after simultaneous treatment with proteasome inhibitor and *p62* or *hsp70* siRNA when compared to pure proteasome inhibitor–treated cells. However, when the cells were exposed to the macroautophagy inhibitor bafilomycin, the silencing of p62 decreased LC3-II levels. In addition, bafilomycin increased p62 accumulation under proteasome inhibition. This relates to the suppression of autophagy. It seems that macroautophagy is a master clearance system to remove proteasome inhibitor–induced perinuclear cargo, but there might be a threshold level for the p62-dependent autophagy induction. The p62 protein seems to regulate the localization of ubiquitinated proteins, since we observed decreased perinuclear accumulation of ubiquitinated proteins in response to *p62* silencing and proteasome inhibition in the RPE cells. However, total ubiquitin levels were not changed in these treatments. A previous study demonstrated that p62 depletion evoked decreased ubiquitination and proteasomal protein degradation [20]. We propose that autophagy is a selective and compensatory clearance system for proteasomes in RPE cells, as has been recently discussed [9,28]. In addition, p62 seems to be an important regulatory protein in the other direction toward proteasomal pathways, since reduced autophagic activity leads to the accumulation of proteasome substrates and this is predominantly regulated by p62 [16]. There is increasing evidence that both p62 and ubiquitin are required for effective protein clearance via proteasomes and autophagy in RPE cells. However, lack of autophagy may lead to the accumulation of p62, which is not good for cells, as it induces a cellular stress response that evokes potentiated cellular damage [38].

All cells and tissues are challenged by different kinds of stresses that vary in their severities and durations. The RPE cells are exposed to chronic oxidative stress; since they are under constant light stimuli, they consume high levels of oxygen and are exposed to the high levels of lipid peroxidation derived from the photoreceptor outer segments. The presence of these stress stimuli means that macromolecules such as proteins are continuously exposed to potential damage that can cause loss of molecular function and depletion of RPE cell populations during aging. One of the most important homeostatic responses involved in maintaining longevity is the induction of heat shock proteins (HSP) to repair damaged intracellular proteins [1,39,40]. Aging and tissue degeneration involve the accumulation of damage in cellular macromolecules in postmitotic cells such as RPE cells. It has been proposed recently that increased protein damage during aging may be exacerbated by a declining heat shock response, reduced levels of HSPs, and the resultant loss of protein quality control [39]. Consequently, sensors such as HSPs that detect different forms of stress have evolved to promote cellular adaptation and survival. The expression of HSPs is induced in response to a variety of chemical and physical stresses to prevent protein damage and detrimental protein aggregation [1,13,41,42].

In addition to these crucial homeostatic functions, recent findings have indicated that HSP70, together with ferritin and metallothionein, chelates lysosomal iron in a non-redox-active form that triggers autophagy clearance [43]. We have demonstrated that oxidative stress strongly induces HSP70 expression in RPE cells [5,44]. There is speculation that oxidative stress may increases permeabilization of lysosomes because of their content of redox-active iron and that may be one reason for the upregulation of the HSPs [45]. HSP70 has been shown to localize within lysosomes and to possess a capacity to depress lysosomal redox-active iron, as well as to modify the permeabilization of lysosomes [13,42,45,46]. In this work, we have demonstrated that p62 strongly colocalizes with proteasome inhibitor–induced perinuclear deposits. There is also strong colocalization between p62 and HSP70. Proteasome inhibitor–induced perinuclear deposits are targeted to macroautophagy. We suppose that p62, rather than HSP70, regulates autophagic activity when studied by the RNA silencing technique. Obviously, HSP70 tries to refold damaged protein, leading to improved vitality in response to proteasome inhibition; however, if this process is not successful, then the proteins are aggregated and targeted to macroautophagic clearance.

Autophagy is attributable to the turnover of damaged cellular components in response to age-related cellular modifications such as starvation, hypoxia, and oxidative stress [1,2]. At present, it is known that autophagic degradation is involved in several human neurodegenerative diseases [14]. Interestingly, it has been demonstrated that in human AMD donor samples there are accumulations of autophagic markers and decreased lysosomal activity [6,7]. An effective autophagic clearance system has also recently been documented in human RPE cells [4,5,7,13]. During aging, lipofuscin accumulates in lysosomes, revealing a decreased cellular capacity to degrade proteins [47]. In addition, lipofuscin promotes the misfolding of intracellular proteins, which evokes additional oxidative stress in RPE cells [48,49]. Once lysosomes are inhibited by bafilomycin, intense autophagosome formation can be observed that coincides with LC3-II accumulation in ARPE-19 cells. If the lysosomal function is suppressed with bafilomycin, macroautophagic clearance is not triggered and proteasome inhibitor–induced protein aggregates are retained in the RPE cell cytoplasm [13]. This might provide a simple chemical model to study lysosomal clearance mechanisms in RPE cells and may help unravel the pathogenesis of AMD, since preservation of the autophagic activity is associated with a lower intracellular accumulation of damaged proteins, better ability to handle protein damage, improvement in tissue function, and retardation of the aging process [14].

Our findings reveal that p62 and HSP70 colocalize strongly in response to proteasome inhibition. Both HSP70 and p62 play central roles in improving cellular viability when proteasomal pathways are disturbed in RPE cells. However, it seems that p62 but not HSP70 can regulate LC3 accumulation in conditions when lysosomes and macroautophagy are inhibited. In proteasome inhibitor–treated RPE cells, the perinuclear protein aggregates undergo effective autophagic clearance. These findings open new avenues for understanding the mechanisms of proteolytic processes in retinal cells, and could be useful in the development of novel therapies targeting p62 and HSP70 [50-52], or the proteins that regulate lysosomal-mediated proteolysis [52,53], with the aim of preventing retinal cell deterioration during aging such as that occurring in AMD [1].
